# Compressive Optic Neuropathy Caused by Orbital Non-Hodgkin's Lymphoma

**DOI:** 10.1155/2012/894062

**Published:** 2012-02-14

**Authors:** Mohammed M. Ziaei, Hadi Ziaei

**Affiliations:** Department of Ophthalmology, Central Middlesex Hospital, Acton Lane, London NW10 7NS, UK

## Abstract

*Purpose*. To present a unique case of Non-Hodgkin's-Lymphoma- (NHL) associated compressive optic neuropathy. *Method*. An 89-year-old male presenting with acute unilateral visual loss and headache. *Results*. Patient was initially diagnosed with occult giant cell arteritis; however after visual acuity deteriorated despite normal inflammatory markers, an urgent MRI scan revealed an extensive paranasal sinus mass compressing the optic nerve. *Conclusion*. Paranasal sinus malignancies occasionally present to the ophthalmologist with signs of optic nerve compression and must be included in the differential diagnosis of acute visual loss.

## 1. Introduction

Paranasal sinus lymphoma is an uncommon malignancy which poses significant diagnostic challenges. Often difficult to diagnose, paranasal mass lesions can potentially cause visual loss by compressing the optic nerve, but only a few such cases have ever been reported in the literature [1–5]. 

## 2. Case Presentation

**Figure 1:**
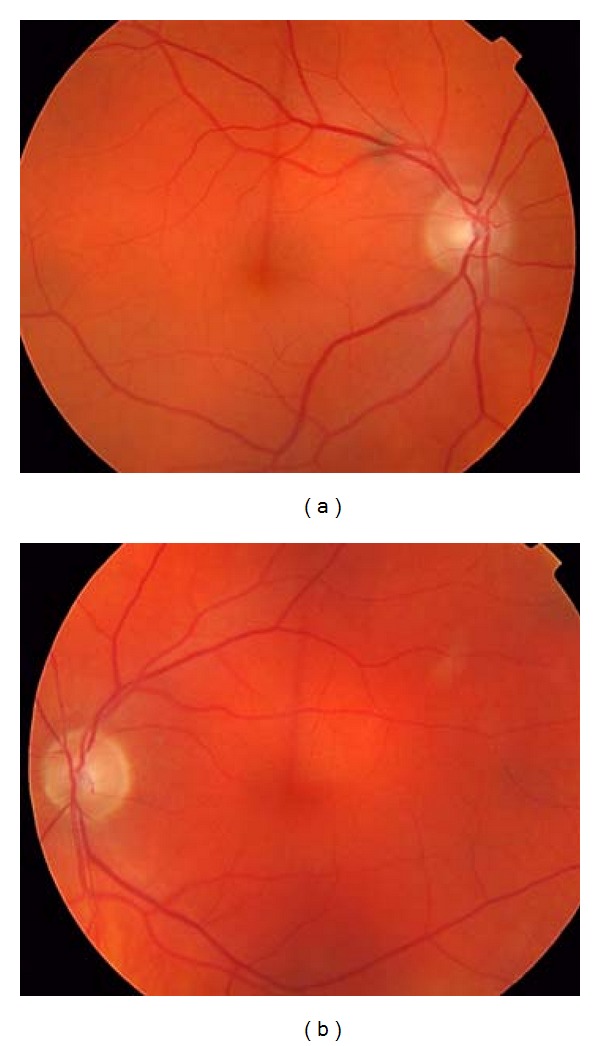
Fundal examination reveals a pale left optic nerve.

An 89-year-old male was seen in the eye clinic with a one-week history of painless visual loss in the left eye along with a recent onset of left-sided headache and malaise. A thorough systemic history failed to reveal any other symptoms. His past ocular history included bilateral pseudophakia whilst he was on medication for hypertension, asthma, and hiatus hernia. Examination revealed a VA of 6/150 (1.36 LOGMAR) in the left eye with reduced colour vision (no colour plates correctly identified on the Ishihara chart). A left relative afferent pupillary defect was detected but neuroophthalmological examination was otherwise unremarkable. Anterior segment examination was normal with clear media and well-centred intraocular lenses. Right fundal examination was unremarkable whilst the left fundus exam revealed pallor of the left optic disc (Figure 1). Fundus fluorescein angiography and macular optical coherence tomography were unremarkable. Left temporal and frontal tenderness was also noted but lymphadenopathy could not be elicited. Blood tests revealed a normal full blood count, electrolytes, ESR (19 mm/hour), and CRP (2.0 mg/L).

A provisional diagnosis of occult giant cell arteritis was made and the patient was started on 1 mg/kg of oral prednisolone. A temporal artery biopsy arranged 5 days later failed to demonstrate any arteritic or granulomatous changes. At this stage a CT of head and orbit was ordered which was reported as showing no orbital or intracranial abnormalities.

**Figure 2:**
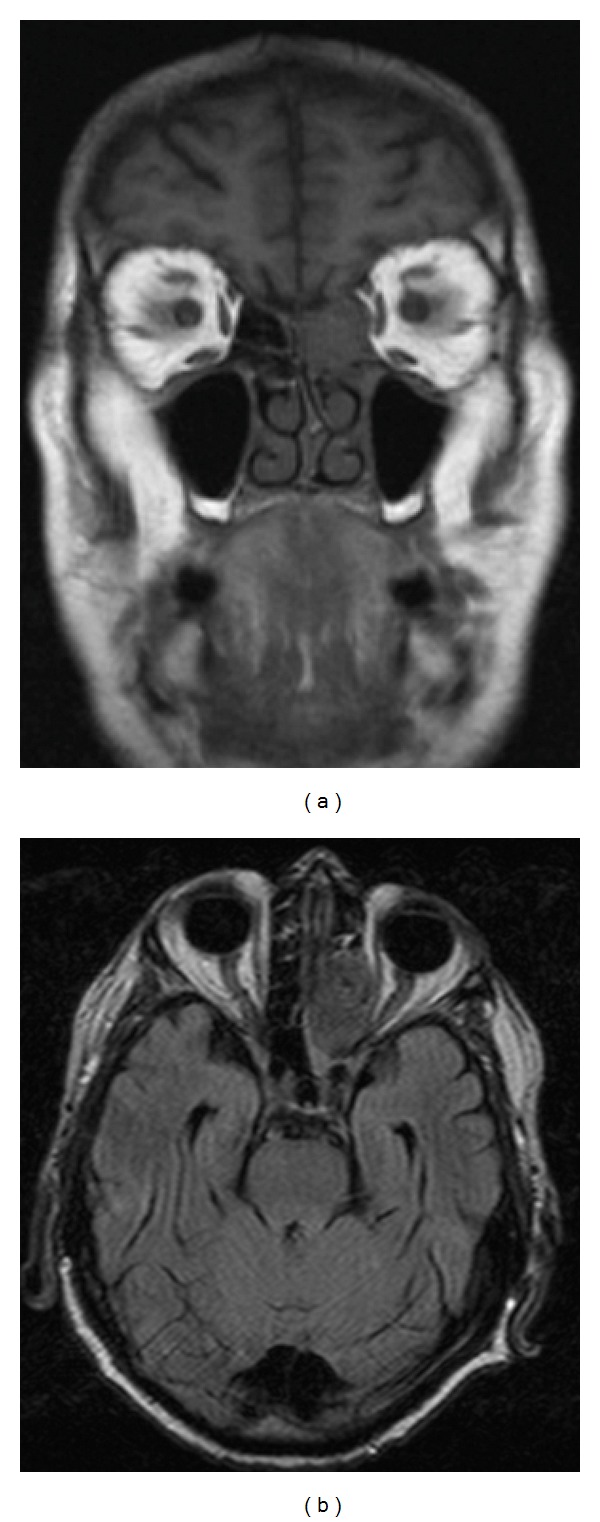
MRI scan shows an abnormal soft tissue lesion within the left paranasal sinuses centered on the ethmoid air cells. Note the distortion of the medial wall of the orbit and narrowing of the orbital apex causing distortion and compression of the left optic nerve.

The patient initially responded well to the corticosteroid treatment with the VA improving to 6/19 (0.5 LOGMAR) a week later. The patient was then monitored and the dose of steroid tapered but the established diagnosis was questioned when the visual acuity declined to 6/60 (1.02 LOGMAR) and the optic disc pallor progressed despite normal inflammatory markers. At this stage a progressive optic neuropathy was suspected and an urgent MRI scan organised which revealed a large soft tissue lesion within the left paranasal sinuses centred on the ethmoidal air cells with narrowing of the medial wall of the orbit causing optic nerve distortion and compression (Figure 2).

An urgent biopsy was arranged with the ENT team which showed aggressive diffuse large B-cell NHL. The patient was referred to a tertiary centre for treatment and has done well with 3 courses of CHOP (cyclophosphamide, doxorubicin, vincristine, and prednisolone) chemotherapy and involved field radiotherapy.

## 3. Discussion

Lymphomas represent a group of malignant neoplasms of lymphoreticular origin which are divided into Hodgkin's disease and NHL [6]. The majority of head and neck NHLs originate from extranodular sites such as lymphoid tissue of Waldeyer's tonsillar ring [7] whilst lymphomas of the nasal cavity and paranasal sinuses are rare, accounting for only 0.17% of all lymphomas [8]. Patients are usually elderly males often with locally advanced tumours [9]. The majority of sinonasal lymphomas are of a diffuse B-cell origin and are classified as stage E1 according to the Ann Arbor system [10, 11].

In one case series the symptoms reported by patients with paranasal sinus lymphoma were nasal obstruction (100%), epistaxis (58%), headaches resistant to analgesia (50%), facial swelling (50%), palatal lesion (42%), and diplopia (17%) [9].

Orbital invasion is a common manifestation of sinus malignancy with frequent presenting signs and symptoms include proptosis, dystopia, visual loss, lid oedema, extraocular movement limitation, diplopia, conjunctival chemosis/injection, eye pain, and photophobia [1].

Treatment options for sinonasal lymphomas include radiation and chemotherapy with some studies showing preference with radiation alone in early disease stages [12, 13], whilst others propose a combined treatment regimen that offers an improved disease-free survival and overall survival rates [14].

Despite treatment paranasal sinus lymphoma carries a poor prognosis (50% 5-year survival rate) often due to a delay in diagnosis as tumors in this area are often asymptomatic initially and are at an advanced stage at the time of diagnosis [1].

Ophthalmological symptoms and signs often occur early in its natural history as a result of the close proximity of the orbital structures to the paranasal sinuses. The ophthalmologist's role therefore is one of upmost importance in recognising the clinical red flags associated with ocular manifestations of this malignancy and in ensuring a multidisciplinary approach in patient investigation, management, and followup.
